# Honokiol improved chondrogenesis and suppressed inflammation in human umbilical cord derived mesenchymal stem cells via blocking nuclear factor-κB pathway

**DOI:** 10.1186/s12860-017-0145-9

**Published:** 2017-08-29

**Authors:** Hao Wu, Zhanhai Yin, Ling Wang, Feng Li, Yusheng Qiu

**Affiliations:** 1 Department of Orthopaedics, The First Affiliated Hospital, College of Medicine, Xi’an Jiaotong University, Xi’an, 710061 People’s Republic of China; 2 Center for Biomedical Engineering and Regenerative Medicine, Frontier Institute of Science and Technology, Xi’an Jiaotong University, Xi’an, 710049 People’s Republic of China

**Keywords:** Osteoarthritis, Honokiol, Interleukin-1β, Mesenchymal stem cell, Cartilage repair

## Abstract

**Background:**

Cartilage degradation is the significant pathological process in osteoarthritis (OA). Inflammatory cytokines, such as interleukin-1β (IL-1β), activate various downstream mediators contributing to OA pathology. Recently, stem cell-based cartilage repair emerges as a potential therapeutic strategy that being widely studied, whereas, the outcome is still far from clinical application. In this study, we focused on an anti-inflammatory agent, honokiol, which is isolated from an herb, investigated the potential effects on human umbilical cord derived mesenchymal stem cells (hUC-MSCs) in IL-1β stimulation.

**Methods:**

Second passage hUC-MSCs were cultured for multi-differentiation. Flow cytometry, qRT-PCR, von Kossa stain, alcian blue stain and oil red O stain were used for characterization and multi-differentiation determination. Honokiol (5, 10, 25, 50 μM) and IL-1β (10 ng/ml) were applied in hUC-MSCs during chondrogenesis. Analysis was performed by MTT, cell apoptosis evaluation, ELISA assay, qRT-PCR and western blot.

**Results:**

hUC-MSC was positive for CD73, CD90 and CD105, but lack of CD34 and CD45. Remarkable osteogenesis, chondrogenesis and adipogenesis were detected in hUC-MSCs. IL-1β enhanced cell apoptosis and necrosis and activated the expression of caspase-3, cyclooxygenase-2 (COX-2), interleukin-6 (IL-6) and matrix metalloproteinase (MMP)-1, −9, 13 in hUC-MSCs. Moreover, the expression of SRY-related high-mobility group box 9 (SOX-9), aggrecan and col2α1 was suppressed. Honokiol relieved these negative impacts induced by IL-1β and suppressed Nuclear factor-κB (NF-κB) pathway by downregulating expression of p-IKKα/β, p-IκBα and p-p65 in dose-dependent and time-dependent manner.

**Conclusions:**

Honokiol improved cell survival and chondrogenesis of hUC-MSCs and inhibited IL-1β-induced inflammatory response, which suggested that combination of anti-inflammation and stem cell can be a novel strategy for better cartilage repair.

**Electronic supplementary material:**

The online version of this article (doi:10.1186/s12860-017-0145-9) contains supplementary material, which is available to authorized users.

## Background

Articular cartilage has limited and insufficient ability to self-regeneration once being damaged. Great efforts have been made consistently so far, whereas, no effective therapeutic approach was claimed to fully repair damaged articular cartilage. Potential therapies based on multi-differentiation characteristics of mesenchymal stem cells (MSCs) and for cartilage regeneration were widely studied recently. MSCs can also secret various growth factors, including transforming growth factor (TGF), insulin-like growth factor (IGF), hepatocyte growth factor (HGF), fibroblast growth factor (FGF) and vascular endothelial growth factor (VEGF), which promoting cell proliferation and angiogenesis in different tissues [1, 2] and preventing cell apoptosis from trauma, oxidation stress, radiation and chemicals [3]. MSCs can be obtained from various mature tissues, such as bone marrow [4], adipose tissue [5], and synovium [6]. Fetus tissues contains abundant MSCs [7], such as umbilical cord blood, placenta [8, 9] and umbilical cord matrix [10]. Human umbilical cord derived mesenchymal stem cell (hUC-MSC) was isolated from human umbilical cord and possess several advantages such as vast source, easy isolation, stable multi-differentiation capacity. However, many studies indicated that the application of MSCs in in vitro osteoarthritis (OA) models led to fibrotic cartilage instead of hyaline cartilage [11], the reasons remained unclear.

Interleukin-1β (IL-1β) is generally regarded as one of the main initiators in OA, which being produced by different types of cell including chondrocytes, macrophages and synovial fibroblasts [12, 13]. Importantly, IL-1β activate inflammatory pathways resulting in a vicious circle of articular cartilage damage rather than cartilage regeneration. Cell survival and chondrogenic potential of MSCs should be negatively affected once being transplanted in such inflammatory environment, but whether anti-inflammation treatment would be an improvement has not been examined. Although anti-inflammation has been studied for decades, significant pathological effects of cytokines on OA still deserve adequate attention.

Anti-inflammation therapy has a long history in OA treatment, but traditional agents, such as dexamethasone and Non-Steroidal Anti-Inflammatory Drugs (NSAIDs), have different adverse effects that limit their long-term application for chronic inflammatory diseases [14, 15]. Honokiol is a natural biphenolic compound purified from a traditional Chinese medicine called *Magnolia officinalis*. Its anti-inflammation function and less adverse effects has been reported [16, 17]. One of the therapeutic targets of honokiol is Nuclear Factor-κB (NF-κB) pathway [18], which regulates important downstream signals in inflammatory process [19]. Thus, honokiol has the potential to be a promising anti-inflammatory drug for different inflammatory diseases including OA.

In this study, we investigated the cell survival and chondrogenesis of hUC-MSCs and the effects of honokiol on hUC-MSCs during chondrogenic process in IL-1β stimulation. Our data may provide useful information and a novel approach for articular cartilage regeneration.

## Methods

### Cell isolation and culture of hUC-MSCs

All human umbilical cords (*n* = 6; gestational ages, 39-40 weeks) were obtained from the First Affiliated Hospital of Xi’an Jiaotong University and the study was approved by the institutional review board of the First Affiliated Hospital of Xi’an Jiaotong University. Human umbilical cords were kept in phosphate buffered saline (PBS) with penicillin in ice-box after collection and delivered to our lab as soon as possible. Then umbilical cords were cut into 5 cm sections and all veins and arteries were removed. After being washed by PBS, sections were minced into 1mm^3^ cubes and placed on a petri dish with the same intervals. These cubes were incubated with Liberase Enzyme Blends™ (Roche, Switzerland) at 37 °C for 30 min to dissolve remaining tissue, then transferred into tubes, cell suspensions were centrifuged at 4 °C and 1500 rpm for 5 min, cells were washed by PBS and re-suspended with α-MEM (Gibco, USA). We counted cell number with a hemocytometer, then cells were cultured on petri dishes with a density of 6 × 10^3^ cells/cm^2^ in incubator at 37 °C and under 5% CO_2_. Culture medium was composed of high glucose α-MEM (Gibco, USA), 10% fetal bovine serum (FBS; Gibco, USA), 100 U/ml penicillin and 0.1 mg/ml streptomycin and replaced twice a week. When cell confluence reached 80%, we used 0.25% trypsin to detach cells from petri dishes and passaged cells at 1: 4 dilutions. Cells were passaged for 3 times, each passage was collected and re-suspended with culture medium. After cell number count, cell density was adjusted to 1 × 10^6^ cells/ml for reservation.

### Multi-differentiation of hUC-MSCs

Cells from 2nd passage were cultured in differentiation culture medium for multi-differentiation (Table. 1) [20]. For chondrogenesis, cells were cultured as pellet. Cells were detached by 0.25% trypsin, then transferred into a 15 ml conical tube for centrifugation at room temperature, 1000 rpm for 5 min. The supernatant was removed and cells were re-suspended with 5 ml chondrogenic differentiation medium and then counted. We adjusted the cell density to 1 × 10^6^ cells/tube and centrifuged cells at room temperature, 1000 rpm for 5 min. The supernatant was discarded and 1 ml chondrogenic differentiation medium was added to each tube, then all tubes were cultured in cell incubator at 37 °C and under 5% CO_2_. For osteogenesis or adipogenesis, each well in 6-well plates was added with 2 ml osteogenic differentiation medium or adipogenic differentiation medium and 200 μl high density cells suspension (a density of 1 × 10^6^ cells/ml), the final cell density in each well was 2 × 10^4^ cells/cm^2^. All groups were cultured for 2 weeks and culture medium was replaced twice a week. At the end of 2nd week, all cells were collected for subsequent analysis.

**Table 1:** Differentiation culture medium

Osteogenesis	Low glucose a-MEM (Gibco, USA) 10% (*v*/v) fetal bovine serum (Gibco, USA) 0.1uM dexamethasone (Sigma-Aldrich, USA) 10 mM β-glycerolphosphate (Sigma-Aldrich, USA) 0.2 mM ascorbic acid (Sigma-Aldrich, USA)
Chondrogenesis	Low glucose a-MEM (Gibco, USA) 0.1 uM dexamethasone (Sigma-Aldrich, USA) 50 μg/mL AsA, 100 μg/mL sodium pyruvate (Sigma-Aldrich, USA) 40 μg/mL proline (Sigma-Aldrich, USA) 10 ng/mL TGF-1 (Invitrogen, USA) 50 mg/mL ITS^+^ premix (Becton Dickinson, USA)
Adipogenesis	Low glucose a-MEM (Gibco, USA) 0.5 mM 3-isobutyl-1-methylxanthine (IBMX, Sigma-Aldrich, USA) 1 μM hydrocortisone (Sigma-Aldrich, USA) 0.1 mM indomethacin (Sigma-Aldrich, USA) 10% rabbit serum (Sigma-Aldrich, USA)

### Flow cytometry

The cells were cultured in high glucose α-MEM (Gibco, USA), 10% FBS (Gibco, USA), 100 U/ml penicillin and 0.1 mg/ml streptomycin for 1 week, culture medium was replaced twice a week. Cells were collected and counted, then re-suspended with α-MEM, we adjusted the cell density at 1 × 10^6^ cells/ml. Each 0.1 ml sample was incubated with 20 μl of CD73-PE, CD90-FITC, CD34-PE, CD45-PE, CD105-PE (Santa Cruz, USA) at 4 °C for 1 h. Antibody binding was analyzed by flow cytometry (Becton Dickson, USA).

### Multi-differentiation staining

After 2 weeks’ culture, cells in osteogenesis and adipogenesis group were fixed with 2.5% glutaraldehyde for 15 min. Cells pellets in chondrogenesis group were fixed with 4% paraformaldehyde for 1 h, then embedded in paraffin and cross sectioned. Slices were stained with hematoxylin and eosin (Beyotime, China). Von Kossa staining (GENMED, China), oil red O staining (Beyotime, China) and alcian blue staining (Beyotime, China) were conducted by using commercial stain kits. All staining results were photographed and analyzed by using microscope (Olympus IX35, Japan).

### qRT-PCR

qRT-PCR were applied to determine gene markers expression in cell differentiation. The total RNAs were extracted from cells by Trizol (Invitrogen, USA) according to the manufacturer’s instructions. 1 μg RNA was used to synthesize cDNA using a reverse transcription reagents kit (Roche, Switzerland). The qRT-PCR was carried out with the following protocol and conducted with Applied Biosystems 7500 Fast (Applied Biosystems, USA). The qRT-PCR system performed with the following temperature profile: 50 °C for 2 min, 95 °C for 2 min, then 40 cycles of 95 °C for 3 s and 40 cycles of 60 °C for 30 s. All data was analyzed by using 2^-ΔΔCT^ method [21]. GADPH was used as control. All primer sequences were showed in Table 2.

**Table 2:** Primer sequences for qRT-PCR

Gene	Forward sequence 5′-3′	Reverse sequence 5′-3’
GADPH	TGTTGCCATCAATGACCCCTT	CTCCACGACGTACTCAGCG
ALP	CCACGTCTTCACATTTGGTG	AGACTGCGCCTGGTAGTTGT
RUNX-2	AGTGGACGAGGCAAGAGTTTC	CCTTCTGGGTTCCCGAGGT
SOX-9	CTTCCGCGACGTGGACAT	GTTGGGCGGCAGGTACTG
Aggrecan	ACAGCTGGGGACATTAGTGG	GTGGAATGCAGAGGT GGTTT
Col2α1	GCCTGGTGTCATGGGTTT	GTCCCTTCTCACCAGCTTTG
CEBP	AGGAACACGAAGCACGATCAG	CGCACATTCACATTGCACAA
FABP4/aP2	TACTGGGCCAGGAATTTGAC	GGACACCCCCATCTAAGGTT
Caspase-3	AGAACTGGACTGTGGCATTGAG	GCTTGTCGGCATACTGTTTCAG
MMP-1	AGTGACTGGGAAACCAGATGCTGA	GCTCTTGGCAAATCTGGCCTGTAA
MMP-9	GCGGAGATTGGGAACCAGCTGTA	GACGCGCCTGTGTACACCCACA
MMP-13	TGCTGCATTCTCCTTCAGGA	ATGCATCCAGGGGTCCTGGC

### Cell viability

Cell viability was evaluated by 3-(4,5- dimethylthiazol-2-yl)-2,5-diphenyltetrazolium bromide (MTT; Sigma-Aldrich, USA). 2nd passage cells were cultured in 96-wells culture plate with a density of 2 × 10^4^ cells/cm^2^. Honokiol (Sigma-Aldrich, USA) was dissolved in dimethyl sulfoxide (DMSO; Sigma-Aldrich, USA) for 24 h in advance and added in cell culture plate with gradient concentration (0, 5, 10, 25, 50 μM). After being washed by PBS, cells were incubated with MTT (0.2 mg/ml) at 37 °C for 4 h. Then culture medium was replaced by DMSO and culture plate was shake for 10 min at room temperature. Each well was determined by microreader (SpectraMax i3, Molecular Devices, USA) at 550 nm.

### ELISA assay for IL-6 and COX-2 production determination

2nd passage cells were cultured with a density of 2 × 10^4^ cells/cm^2^ in 6-wells plate with chondrogenic medium, control group was cultured in common culture medium. IL-1β group was treated with IL-1β (10 ng/ml; Sigma-Aldrich, USA), honokiol group were treated with both honokiol (5, 10, 25 μM) and IL-1β (10 ng/ml) for 24 h. IL-6 and COX-2 production was determined by using commercial ELISA kit (R&D System, USA). All plates were read at 460 nm by a microreader (SpectraMax i3, Molecular Devices, USA).

### Inflammatory stimulation and honokiol treatment

2nd passage hUC-MSCs were randomly divided into 4 groups, control group was cultured in common medium. Remaining groups were cultured as pellets for chondrogenesis, the method was previously described. IL-1β group was treated with IL-1β (10 ng/ml), honokiol group was treated with both IL-1β (10 ng/ml) and honokiol (25 μM). Chondrogenesis group was cultured in chondrogenic medium without any additions. All groups were cultured for 2 weeks and culture mediums were replaced twice a week. At the end of 2nd week, cells were collected for subsequently analysis.

### Apoptosis analysis

Cell apoptosis and necrosis of hUC-MSCs were assessed by Hoechst 33,342 and propidium iodide (PI) staining using a commercial kit (Beyotime, China) and evaluated by fluorescence microscopy (Olympus IX35, Japan). Results were analyzed by ImageJ (NIH, USA).

### Immunofluorescent staining

All slides were rinsed with PBS, and fixed with 2.5% glutaraldehyde for 15 min, followed by three times PBS washing, then treated with 0.3% Triton X-100 for 0.5 h and 1% BSA in PBS for 1 h. Rabbit anti-collagen II monoclonal antibody (1:100, Abcam, UK) was incubated at 4 °C overnight, followed by Alexa flour 488 conjugated secondary antibody (Molecular Probes, USA) incubation at 37 °C for 1 h. Cells were counterstained with DAPI for 10 min and analyzed by fluorescence microscopy (IX53, Olympus, Japan).

### Western blot analysis

To investigate the pattern of suppressive effects of honokiol on p-IKKα/β, p-IκBα, p-p65, 2nd passage hUC-MSCs were grouped as previously described in inflammatory stimulation part and treated in two different ways. One is that honokiol group was cultured with gradient dose of honokiol (5, 10, 25 μM) and IL- β (10 ng/ml) for 2 weeks. The other one is that honokiol group was cultured with honokiol (25 μM) and IL- β (10 ng/ml) for 1, 3, 7 and 14 days respectively. We determined the expression of p-IKKα/β, p-IκBα, p-p65 at each preset time point using western blot, the protocol was briefly described as followed: cells were detached by 10% trypsin and washed by PBS, then lysed by lysis buffer containing protease inhibitors (TianGen, China). The total protein concentration was determined by the Bicinchoninic acid assay (BCA assay; Bio-Rad, USA). Protein extracts were heated for denaturation at 100 °C for 5 min and a 12% sodium dodecyl sulfate polyacrylamide gel electrophoresis (SDS-PAGE; Bio-Rad, USA) was used for electrophoretic separation of proteins. Proteins were transferred to a PVDF membrane (Millipore, USA). The membrane was blocked with 5% non-fat dried milk in TBST buffer (0.1 M Tris-HCl and 0.1% Tween-20, pH = 7.5) for 1 h and probed with β-actin (1: 1000, Santa Cruz Biotechnology, USA), p-IKKα/β, p-IκBα, p-p65 (1: 500, Santa Cruz Biotechnology, USA), Horseradish peroxidase-conjugated anti-rabbit was used as the secondary antibody (1: 1000, Jackson Immunoresearch, USA). The detection was performed by the Thermo-Scientific Pierce ECL Western blotting substrate (Thermo-Fisher Scientific, USA). Images were scanned by Tanon-410 automatically gel imaging system (Shanghai Tianneng Corporation, China), all samples were normalized to the internal control β-actin and the optical density were determined by ImageJ (NIH, USA).

### Statistical analysis

Cells obtained from 6 donors were made into a mixture for this study. All results were presented as mean ± SD and analyzed with two-tailed Student’s *t* –test and one-way analysis of variance (ANOVA). Statistical analysis was conducted by SPSS 23.0 for Mac (IBM Inc., USA); *p* < 0.01 was considered as significant.

## Results

### Flow cytometry

**Fig. 1:**
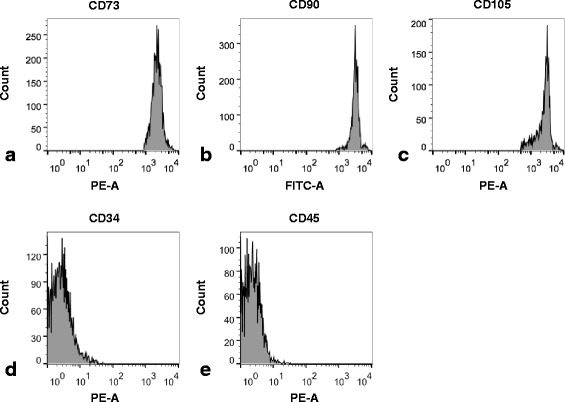
Flow cytometry analysis of cell surface marker expression on hUC-MSCs. hUC-MSCs were cultured in high glucose α-MEM containing 10% FBS, 100 U/ml penicillin and 0.1 mg/ml streptomycin and were passaged for two times. Then cells were analyzed by flow cytometry (*n* = 3). The data presented here were representative of all sample obtained in 6 donors. **a**: CD73, **b**: CD90, **c**: CD105, **d**: CD34, **e**: CD45

The cell surface markers for MSCs were analyzed by flow cytometry, the results showed that cells expressed high levels of CD73, CD90 and CD105, but were lack of CD34 and CD45 expression (Fig. 1).

### Histochemical staining

**Fig. 2:**
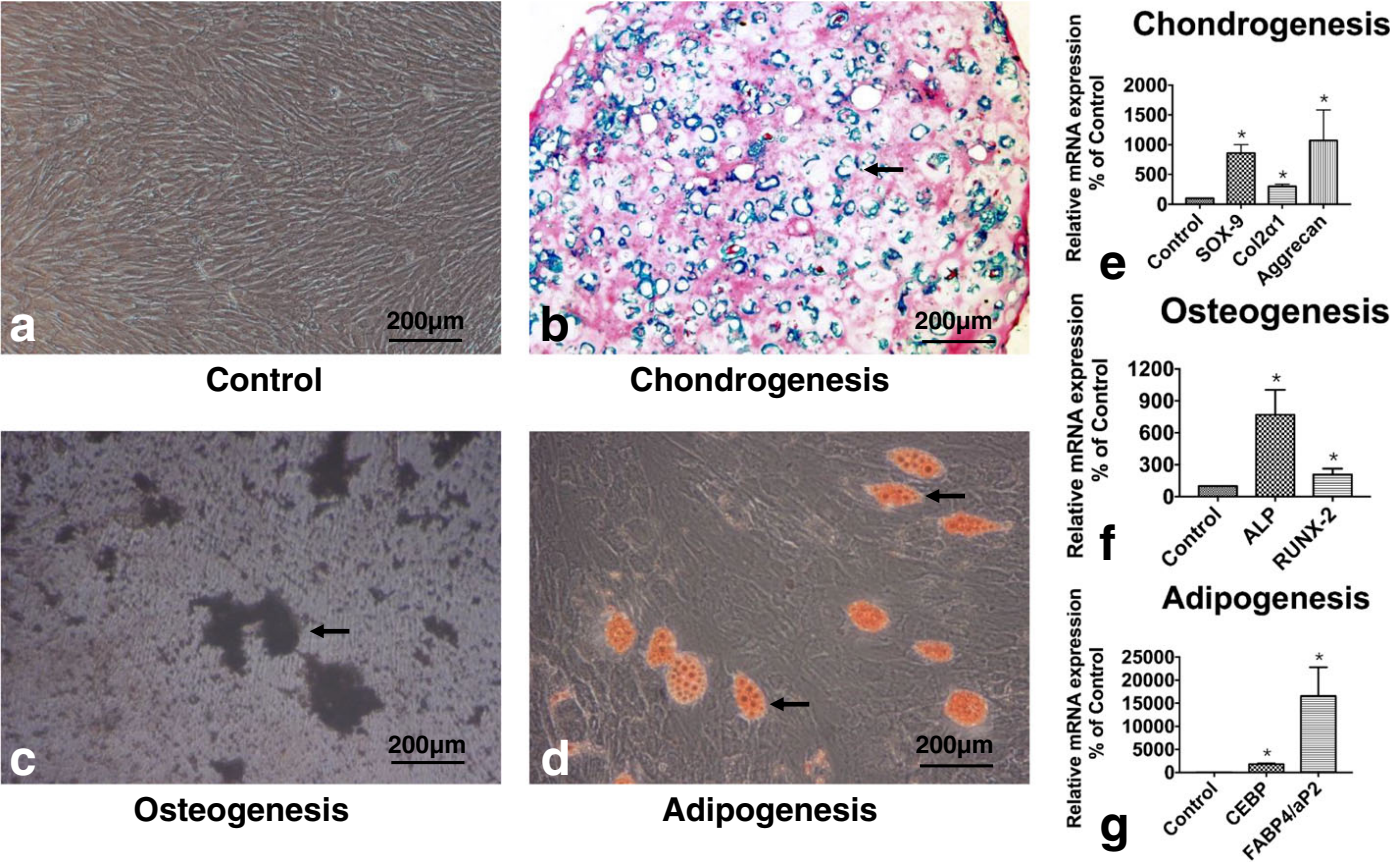
Determination of multi-differentiation of hUC-MSCs. 2nd passage hUC-MSCs were used for multi-differentiation determination. For osteogenesis or adipogenesis, cells were cultured in 6-wells plate with osteogenic or adipogenic medium for 2 weeks. Chondrogenesis was conducted in pellet culture for 2 weeks (*n* = 6). **a**: control group. **b**: Chondrogenesis group cells. Hematoxylin-eosin and alcian blue dual-staining (arrow indicated the positive alcian blue staining). c: Osteogenesis group cells (arrow indicated the positive von Kossa staining). **d**: Adipogenesis group cells (arrow indicated the positive oil red O staining). The results showed here were representative of all samples from 6 donors. The expression of differentiation related genes was evaluated by qRT-PCR. **e**: Chondrogenesis related genes. f: Osteogenesis related genes. g: Adipogenesis related genes. Data was analyzed by using the 2^-ΔΔCT^ method. All qRT-PCR results were presented as mean ± SD (*n* = 9); p* < 0.01 versus control

After 2 weeks’ culture, each differentiation group was evaluated by alcian blue staining, oil red O staining and von Kossa staining respectively. Our results showed vary degrees of positive staining results in differentiation groups. Control group cells remained the shape of MSCs with negative staining marks (Fig. 2a), In chondrogenesis group, cells were cultured in pellets, positive alcian blue staining was detected in slices (Fig. 2b). In osteogenesis group, von Kossa stain-positive nodules were formed (Fig. 2c). In adipogenic group, positive oil red O stain-cells were widely detected (Fig. 2d).

### qRT-PCR

We also evaluated key genes of differentiation in hUC-MSCs. Our results revealed that the expression of SOX-9, col2α1, aggrecan in chondrogenesis group was 8.6 (*p* = 9.65 × 10^−5^), 3 (*p* = 2 × 10^−3^) and 10 (*p* = 6.4 × 10^−4^) folds of control respectively (Fig. 2e), The expression of osteogenic markers, Alkaline phosphatase (ALP) and Runt-related transcription factor 2 (RUNX-2) in osteogenesis group had approximate 7.7 (*p* = 6.79 × 10^−4^) and 2 (*p* = 2.26 × 10^−4^) folds of control (Fig. 2f). Additionally, the expression of adipogenic markers, CCAAT-enhancer-binding proteins (CEBP) and fatty acid-binding protein-4 (FABP4/aP2) in adipogenesis group, were highly expressed and had almost 18.3 (*p* = 5.83 × 10^−6^) and 165 (*p* = 2.02 × 10^−5^) folds of control separately (Fig. 2g).

### The effects of honokiol on cell viability in hUC-MSCs

**Fig. 3:**
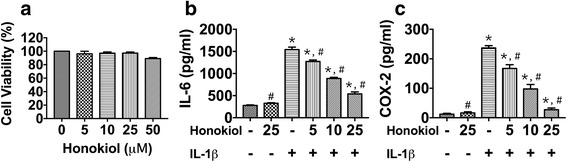
Cell viability and the production of IL-6 and COX-2 in a gradient dose of honokiol. 2nd passage hUC-MSCs were cultured in high glucose α-MEM containing 10% FBS, 100 U/ml penicillin and 0.1 mg/ml streptomycin. A gradient dose of honokiol (0, 5, 10, 25, 50 μM) with or without IL-1β (10 ng/ml) were added for incubation for 24 h. **a**: Cell viability determination. **b**, **c**: IL-6 and COX-2 production. All results were presented as mean ± SD (*n* = 6); p* < 0.01 versus control; *p*# < 0.01 versus IL-1β alone (10 ng/ml IL-1β without honokiol)

MTT assay was introduced here to investigate the effect of different concentration of honokiol on cell viability. The results showed that the gradient dose (5, 10, 25, 50 μM) of honokiol didn’t have remarkable cytotoxic effect on hUC-MSCs until the concentration increased to 50 μM (Fig. 3a). During the culture of hUC-MSCs, SOX-9, Aggrecan and Col2α1 expression were evaluated in different passages of cells to investigate the prime cell passage for chondrogenesis, the results indicated that three markers were highly expressed in the 2nd passage cells (Additional file 1: Figure S1).

### The effects of honokiol on IL-6 and COX-2 production in hUC-MSCs

According to previous results, the relatively safe dose of honokiol in vitro ranged from 5 to 25 μM. To discover the prime dose of honokiol for anti-inflammation in vitro, hUC-MSCs were treated with honokiol (5, 10, 25 μM) under IL-1β (10 ng/ml) stimulation for 24 h. The ELISA results showed that honokiol suppressed IL-6 and COX-2 production in hUC-MSCs in a dose-dependent way (Fig. 3b/c). Especially, IL-6 and COX-2 production in honokiol group was still higher than control group, but production in honokiol group (25 μM) were only 34.9% (*p* = 2.94 × 10^−11^) and 11.6% (*p* = 2.42 × 10^−13^) of IL-1β group respectively.

### Cell survival of hUC-MSCs in IL-1β stimulation

**Fig. 4:**
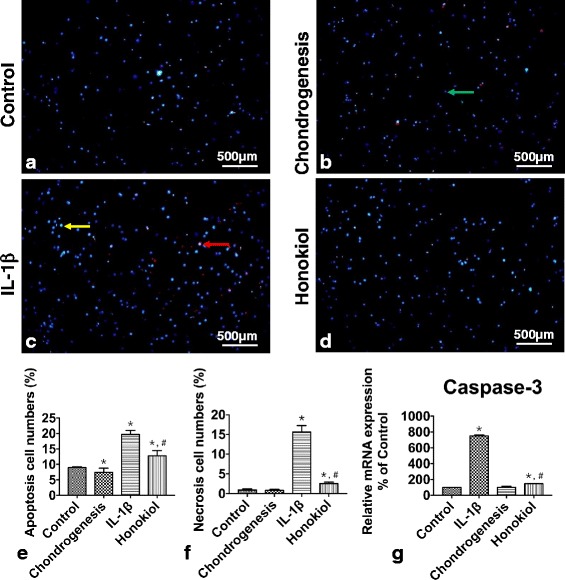
The effects of honokiol on apoptosis and necrosis of hUC-MSCs. 2nd passage hUC-MSCs were cultured in 4 groups, **a**: Control, **b**: Chondrogenesis, **c**: IL-1β, **d**: Honokiol for 2 weeks. At the end of 2nd week, cells were stained with Hoechst 33,342 and PI for apoptosis and necrosis analysis. Green arrow indicated the normal cell, yellow arrow indicated the apoptotic cell and red arrow indicated the necrotic cell. The apoptotic cell number and necrotic cell number were analyzed by ImageJ, all data were presented as mean ± SD (*n* = 3), p* < 0.01 versus control; *p*# < 0.01 versus IL-1β. **e**: apoptotic cells percentage, **f**: necrotic cells percentage. Caspase-3 expression was determined by qRT-PCR. **g**: caspase-3 expression. Gene expression data were analyzed by using the 2^-ΔΔCT^ method. All qRT-PCR results were presented as mean ± SD (*n* = 9); p* < 0.01 versus control; *p*# < 0.01 versus IL-1β

25 μM was selected for applied concentration of honokiol in subsequent experiments according to our study. A commercial fluorescence staining kit was used to evaluate cell apoptosis and necrosis. Control and chondrogenesis group cells showed low degree of apoptosis and necrosis (Fig. 4a/b), which had 8.99% apoptotic cells and 0.91% necrotic cells in control group and 7.47% and 0.82% in chondrogenesis group (Fig. 4e/f). Cell apoptosis and necrosis were enhanced in IL-1β group (Fig. 4c), the apoptotic and necrotic cells were 19.71% (*p* = 3.85 × 10^−9^) and 15.63% (*p* = 3.72 × 10^−9^) in IL-1β group compared with the low percentage in control and chondrogenesis group (Fig. 4e/f). Apoptotic and necrotic cell percentages were improved in honokiol group, which were 12.75% (*p* = 9.6 × 10^−6^) and 2.5% (*p* = 1.96 × 10^−8^) respectively (Fig. 4d) and much lower than IL-1β group (Fig. 4e/f). qRT-PCR was used for evaluating the expression of caspase-3 (Fig. 4g), which is one of the most important regulators and markers for apoptosis. Results showed that the expression of caspase-3 in IL-1β group was 7.5 folds of control (*p* = 5.15 × 10^−9^), chondrogenesis group had an equal level with control (*p* = 0.72361). In honokiol group, the expression were only 1.45 folds of control (*p* = 1.28 × 10^−4^) and 19% of IL-1β group (*p* = 3.06 × 10^−9^).

### Maintenance of Chondrogenic potential of hUC-MSCs

**Fig. 5:**
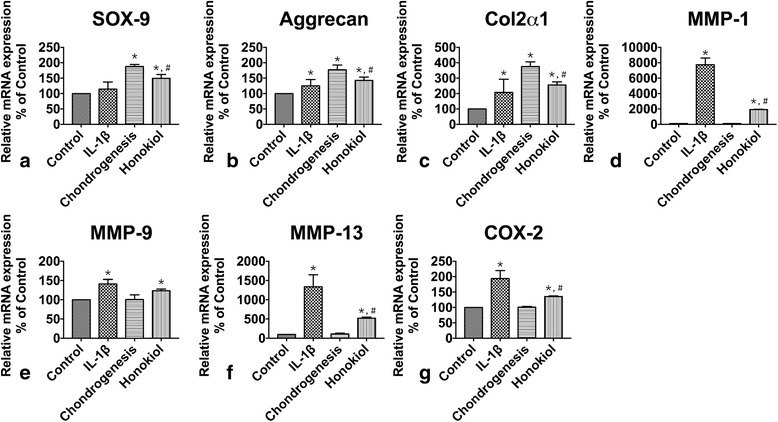
The effects of honokiol on expressions of Caspase-3, SOX-9, Aggrecan, Col2α1, MMP-1, MMP-9, MMP-13 and COX-2. 2nd passage hUC-MSCs were cultured in pellets with chondrogenic medium, cells were treated with IL-1β (10 ng/ml) and honokiol (25 μM). After 2 weeks, Gene expression was evaluated by qRT-PCR. **a**: SOX-9, **b**: Aggrecan, **c**: Col2α1, **d**: MMP-1, **e**: MMP-9, **f**: MMP-13, **g**: COX-2. Data was analyzed by using the 2^-ΔΔCT^ method. All results were presented as mean ± SD (*n* = 9); p* < 0.01 versus control; *p*# < 0.01 versus IL-1β alone (10 ng/ml IL-1β without honokiol)

**Fig. 6:**
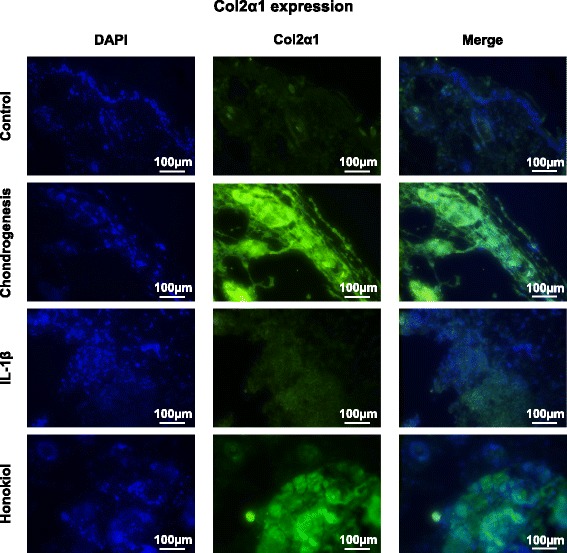
Expression of Col2α1. Immunofluorescent staining results of Col2α1 were representative of the cells obtained in 6 donors. DAPI stain (blue) indicated the cells, col2α1 stain (green) indicated the positive expression of col2α1 (*n* = 3)

Chondrogenic potential of hUC-MSCs is the key for cartilage regeneration, especially in IL-1β stimulation. As described previously, SOX-9, aggrecan and col2α1 were selected as markers for chondrogenesis and cartilage ECM formation (Fig. 5a/b/c). In chondrogenesis group, elevations of expression in SOX-9, aggrecan and col2α1 were noticed, which were 1.8-. 1.7-, 3.7-fold of control respectively (*p* = 1.6 × 10^−5^, 6.1 × 10^−4^, 1.3 × 10^−5^). However, the expression level was 1.1-, 1.2- and 2-fold of control (*p* = 0.37067, 0.27118, 0.04465) in IL-1β group. The expressions were upregulated to varied extent in honokiol group, which were 1.3-, 1.1-, 1.3- fold of IL-1β group respectively (*p* = 9.604 × 10^−3^, 8.0956 × 10^−3^, 3.363 × 10^−3^). Immunofluorescent staining was also used for the evaluation of col2α1 expression in different groups. The results indicated that the expression of col2α1in control and IL-1β group remained a low level compared with chondrogenesis group and honokiol group, moreover, the expression in honokiol was lower than in chondrogenesis group (Fig. 6).

### Cartilage ECM degradation and inflammation activation of hUC-MSCs

ECM is the main content of cartilage and its degradation is enhanced by cell apoptosis and inflammation. In cartilage repair, ECM provides a suitable environment for cells to proliferate and differentiate. MMPs play a very important role in ECM degradation in many cell types. MMP-1, −9, −13 were assessed in our study (Fig. 5d/e/f). Results showed that the expression of MMP-1, −9, −13 in IL-1β group was highly elevated, which were 77-, 1.4- and 13-fold of control respectively (*p* = 2.8 × 10^−6^, 2.15 × 10^−3^, 1.1 × 10^−4^). The expression was almost at the same level in control and chondrogenesis group (*p* = 0.12229, 0.97183, 0.33852). Expressions were inhibited in honokiol group, which were only 24%, 87% and 39% of IL-1β group (*p* = 2.8 × 10^−5^, 0.07168, 6.21 × 10^−3^). COX-2 is the central regulators and effectors in inflammation relating to many clinical symptoms in OA (Fig. 5g), result showed no statistical difference in expression of COX-2 between control and chondrogenesis group (*p* = 0.30602), but expression was 1.9-fold of control (*p* = 1.22 × 10^−3^) in IL-1β group, but had a 30% decline in honokiol group compared with IL-1β group (*p* = 1.6 × 10^−6^).

### Honokiol inhibited NF-κB pathway activation in hUC-MSCs

**Fig. 7:**
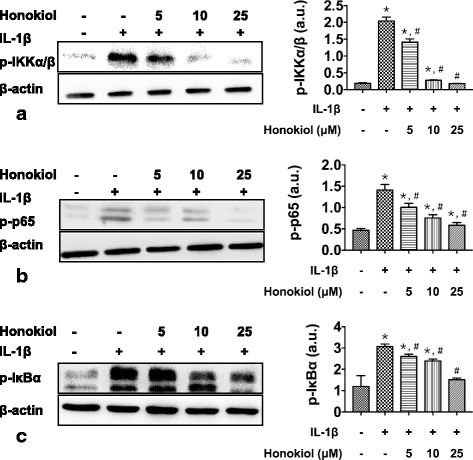
Honokiol inhibited NF-κB pathway in hUC-MSCs in a dose-dependent manner. 2nd passage hUC-MSCs were cultured in pellets with chondrogenic medium, cells were treated with IL-1β (0 or 10 ng/ml) and honokiol (0, 5, 10, 25 μM). After 2 weeks, the expression of (**a**): p-IKKα/β, (**b**): p-p65, (c): p-IκBα were evaluated by western blot. Integrated density values were analyzed by ImageJ and normalized to β-actin, all results were presented as mean ± SD (*n* = 9); p* < 0.01 versus control; *p*# < 0.01 versus IL-1β alone (10 ng/ml IL-1β without honokiol)

**Fig. 8:**
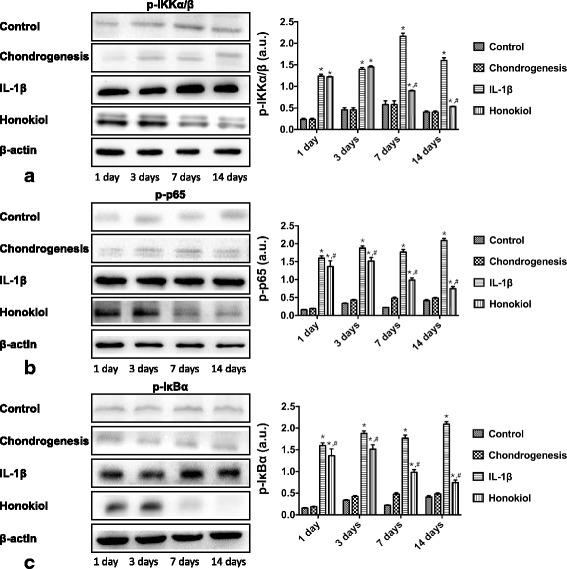
Honokiol inhibited NF-κB pathway in hUC-MSCs in a time-dependent manner. 2nd passage hUC-MSCs were cultured in pellets with chondrogenic medium, cells were treated with IL-1β (10 ng/ml) and honokiol (25 μM). At 1st day, 3rd day, 7th day and 14th day, the expression of (a): p-IKKα/β, (b): p-p65, (c): p-IκBα were evaluated by western blot. Integrated density values were analyzed by ImageJ and normalized to β-actin, all results were presented as mean ± SD (*n* = 9); p* < 0.01 versus control; *p*# < 0.01 versus IL-1β alone (10 ng/ml IL-1β without honokiol)

NF-κB pathway is a significant cellular and molecular regulative network in inflammatory diseases, whose effects on OA initiation and progression can’t be underestimated. High expression of p-IKKα/β, p-IκBα and p-p65 is applicative marker for NF-κB pathway activation. The western blot results demonstrated that honokiol suppressed the expression of p-IKKα/β, p-IκBα and p-p65 in a dose-dependent way, the expression of p-IKKα/β, p-IκBα and p-p65 in hUC-MSCs dropped along with the increase of honokiol concentration (Fig. 7a/b/c). Moreover, during 2 weeks chondrogenesis, the expression of p-IKKα/β, p-IκBα and p-p65 in IL-1β group remained a high level in contrast to control and chondrogenesis group. With a slight upregulation in first 3 days, the expression of p-IKKα/β, p-IκBα and p-p65 in hUC-MSCs declined from the 3rd day, which indicating that honokiol suppressed the expression of p-IKKα/β, p-IκBα and p-p65 in a time-dependent manner in long-term application (Fig. 8a/b/c).

## Discussion

OA is an inflammatory disease and characterized with pain and cartilage degradation. Pro-inflammation cytokines, such as IL-1β, play important roles in different stages of OA. Anti-inflammation is considered as one effective therapy for OA symptoms, but cartilage repair or cartilage regeneration is the fundamental solution for OA. Stem cell-based therapeutic strategy for cartilage repair is widely studied in recent years. Various types of stem cell were considered as the promising candidates for cartilage regeneration. hUC-MSCs have its advantages such as vast source and easy isolation among them. However, the stem cell-based cartilage regeneration didn’t achieve a satisfied therapeutic effect in different studies [11]. Anti-inflammation is the main therapy for OA in clinical practice, however, the combination of stem cell-based therapy and anti-inflammation remains a new concept for cartilage regeneration. Despite of various traditional anti-inflammation agents, honokiol, which is an extract from traditional Chinese medicine, was introduced in this work. Anti-inflammation, anti-oxidation and other pharmacological features were reported recently [18, 22], thus, honokiol may prove to be a potential candidate for OA therapy. Studies indicated that MSCs being applied in OA models usually formed fibrotic cartilage instead of hyaline cartilage [11]. Therefore, it is easy to hypothesize that inflammation has the negative effect on MSCs once being transplanted in an inflammatory environment, inflammation may be one of major reasons for the unsuccessful MSCs-based cartilage repair. In our study, we demonstrated that IL-1β suppressed the cell survival, chondrogenesis and ECM degradation in hUC-MSCs, but honokiol relieved the negative effects by partly blocking the activation of NF-κB pathway. These findings reported here are the first of this kind of study according to our knowledge. It has been well-documented that OA joints express high level of pro-inflammatory cytokines, such as IL-1β and TNF-α [13], our data indicated anti-inflammation and protective effect of honokiol on hUC-MSCs may provide a novel thought for stem cell-based cartilage repair. In addition, hUC-MSCs were positive for certain cell surface markers of MSCs and performed successful chondrogenesis, osteogenesis and adipogenesis. The fulfillment of the criteria for stem cell therapy [23] ensured the isolation of MSCs from human umbilical cords.

IL-1β is one of the pro-inflammatory cytokines getting involved with various inflammatory diseases. IL-1β is highly expressed in damaged articular cartilage during the initiation and progression of OA, the detection of IL-1β in synovium and articular fluid had been noticed as well [24]. IL-1β can stimulate COX-2 to upregulate other inflammatory mediators. Other pro-inflammatory cytokines, such as IL-6, also can be upregulated by IL-1β [13]. IL-1β activates the expression of MMPs contributing to ECM degradation of cartilage [25]. The relevance of IL-1β and elevated level of human chondrocyte apoptosis, enhances the synthesis of aggrecanase in human chondrocytes and synovial fibroblasts during OA was also reported [26]. The inhibition of synthesis of col2α1 and proteoglycan by IL-1β results in catabolism of cartilage tissue [27]. Our study indicated that honokiol inhibited the production of IL-6 and COX-2 in a dose-dependent manner in hUC-MSCs. Moreover, IL-1β can bind to IL-1 receptor and other receptors, then activate downstream cellular and molecular signals such as NF-κB pathway and Mitogen-activated protein kinases (MAPK) pathway to induce the cascades activation of caspases, which is the key process in apoptosis. Caspase-3 acts as the effector in apoptosis. We reported that IL-1β induced a significant apoptosis and necrosis in hUC-MSCs, the expression of caspase-3 was highly up-regulated in this process. Apoptosis and necrosis were remarkably inhibited by honokiol, which was clearly presented in Fig. 4.

Chondrogenesis is a sophisticated process being associated with many genes and cellular signals. SOX-9, an important regulator in the early stage of chondrogenesis, can regulate downstream genes related to chondrogenesis and ECM synthesis, such as col2α1 and aggrecan [28–31]. In normal chondrogenic process, MSCs differentiate into progenitor cells, then into chondrocytes, synthesizing col2α1 and aggrecan, but in some cases, especially inflammation, normal chondrocytes undergo hypertrophy leading to the production of collagen type I and XI, which are main ECM contents of fibrotic cartilage [32, 33]. IL-1β suppressed the expression of SOX-9, aggrecan and col2α1 according to our results, which indicated an unsatisfied chondrogenesis of hUC-MSCs. Instead, the expression of the three proteins were up-regulated by honokiol and recovered to some extent, honokiol showed the capacity of maintaining the chondrogenic potential of hUC-MSCs in some ways.

Cartilage ECM provide the fundamental support for cell survival, proliferation and chondrogenesis of hUC-MSCs. Aggrecan and col2α1 are two main components of ECM in hyaline cartilage, both can be inhibited by IL-1β as we described previously. IL-1β not only suppresses ECM synthesis, but also enhances ECM degradation through activation of certain proteins. The main enzymes responsible for ECM degradation are MMPs, a large protein family containing various proteases. MMP-1, 9, 13 were assessed and showed an excessive synthesis in IL-1β group, but the upregulation was partly blocked by honokiol. The inhibition of MMP-9 wasn’t as effective as MMP-1, 13 by honokiol. Interestingly, the substrates of MMP-1 include collagen type I, II, III, VII, VIII, X and gelatin, as to MMP-13, collagen type IV, IX, XIV are also included. However, the substrates of MMP-9 only contained gelatin, collagen type IV and V [34]. Thus, MMP-1, 13 may serve a more crucial role than MMP-9 in cartilage degradation as the main collagen type in articular cartilage ECM is collagen type II. It was indicated that honokiol was an appropriate anti-inflammation agent for hUC-MSCs to survive and differentiate in IL-1β stimulation.

The inhibition of NF-κB pathway is one vital process in anti-inflammation of honokiol, which has been proved in several types of mature cells. NF-κB pathway regulates various cellular signals in inflammation and has great therapeutic significance in OA [19], p-IKKα/β, p-IκBα and p-p65 are the phosphorylated forms of three important proteins in NF-κB pathway. Once NF-κB is activated by the binding of IL-1β and its receptors, these proteins are phosphorylated and translocated from cytoplasm to nucleus. The high level of the protein phosphorylation is a symbol for NF-κB activation. Our study reported that p-IKKα/β, p-IκBα and p-p65 were elevated by IL-1β stimulation as expected and the expression of p-IKKα/β, p-IκBα and p-p65 in hUC-MSCs were suppressed by honokiol in both dose-dependent and time-dependent manner. As the concentration of IL-1β applied in our study was much higher than in OA joint, the anti-inflammation and protective effects of honokiol on hUC-MSCs may be more efficient in long-term in vivo application with an accurate and sustained release-control of honokiol. OA is characterized with chronic pathological process and low concentration of IL-1β, our colleagues are focusing on an animal model to test the hypothesis and preliminary data will be published in near future.

## Conclusions

The expectation of regenerating or reconstructing cartilage defect by simple application of MSCs is proved less effective. One possible reason is the intensive focus on stem cells (the seeds) but ignoring cell living environment (the soil). In brief, IL-1β induced apoptosis in hUC-MSCs followed by ECM degradation, synthesis down-regulation, inflammation activation and cytokines secretion. We demonstrated that honokiol can significantly improve cell survival, maintain chondrogenic potential and ECM synthesis in hUC-MSCs by inhibiting NF-κB activation in dose-dependent and time-dependent way. Given the complex inflammation regulatory networks, cell survival, chondrogenesis and ECM production in hUC-MSCs didn’t recover or maintain at the normal level, however, the combination of anti-inflammation and hUC-MSCs may be a novel strategy for cartilage regeneration. In vitro study was only a primary verification of our hypothesis, more attention has been focused on sustained-release of anti-inflammatory agent, MSCs and tissue engineering scaffolds to construct a cartilage regenerative complex. These findings will provide a new thought for cartilage repair.

## Additional file

Additional file 1: Figure S1. Prime passage number of hUC-MSCs. hUC-MSCs from 1st, 2nd, 3rd passage were cultured in chondrogenic medium as pellets for 2 weeks. The expression of SOX-9, col2α1 and aggrecan was evaluated by qRT-PCR. Data was analyzed by using the 2^-ΔΔCT^ method. All results were presented as mean ± SD (*n* = 9); *p** < 0.01 versus P1. (PPTX 206 kb)

## Abbreviations

ALP: Alkaline phosphatase
CEBP: CCAAT-enhancer-binding proteins
COX-2: Cyclooxygenase-2
ELISA: Enzyme linked immunosorbent assay
FABP4/aP2: Fatty acid-binding protein-4
FGF: Fibroblast growth factor
GAPDH: Glutaraldehyde phosphate dehydrogenase
HGF: Hepatocyte growth factor
hUC-MSCs: Human umbilical cord derived messechymal stem cells
IGF: Insulin-like growth factor
IL-1β: Interleukin-1β
IL-6: Interleukin-6
IκBα: Inhibitor of nuclear factor κB α
MMP: Matrix metalloproteinase
MSCs: Mesenchymal stem cells
NF-κB: Nuclear factor-κB
OA: Osteoarthritis
PBS: Phosphate buffered saline
p-IKKα/β: Inhibitor of nuclear factor κB kinase α/β
qRT-PCR: Quantitative real-time polymerase chain reaction
RUNX-2: Runt-related transcription factor 2
SOX-9: SRY-related high-mobility group box 9
TGF: Transforming growth factor
VEGF: Vascular endothelial growth factor
