# Supplementary figures and images for: Honokiol improved chondrogenesis and suppressed inflammation in human umbilical cord derived mesenchymal stem cells via blocking nuclear factor-κB pathway
Source: BMC Cell Biol. 2017 Aug 29;18:29. doi: 10.1186/s12860-017-0145-9 (PMC5576244; doi:10.1186/s12860-017-0145-9)

## Slide 1
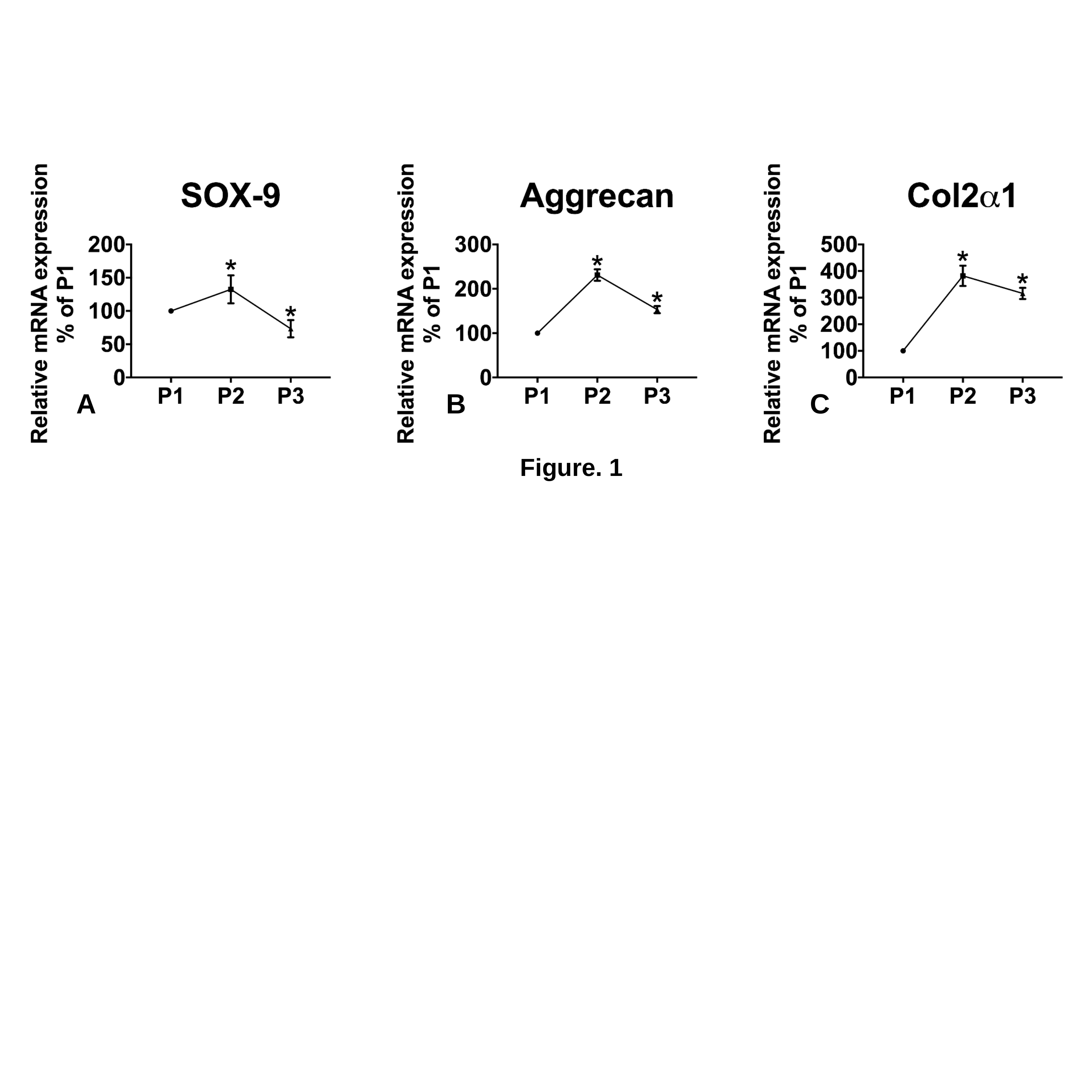

A
B
C
Figure. 1

Supplement: Additional file 1: Figure S1. — Prime passage number of hUC-MSCs. hUC-MSCs from 1st, 2nd, 3rd passage were cultured in chondrogenic medium as pellets for 2 weeks. The expression of SOX-9, col2α1 and aggrecan was evaluated by qRT-PCR. Data was analyzed by using the 2-ΔΔCT method. All results were presented as mean ± SD (n = 9); p* < 0.01 versus P1. (PPTX 206 kb) [file 12860_2017_145_MOESM1_ESM.pptx]
